# Urinary infection due to *Balantioides coli*: a rare accidental zoonotic disease in an addicted and diabetic young female in Iran

**DOI:** 10.1099/jmmcr.0.000102

**Published:** 2016-02-05

**Authors:** Saman Soleimanpour, Ali Babaei, Abdolghayyoum Movahhedi Roudi, Seyedeh Sara Raeisalsadati

**Affiliations:** ^1^​ Student Research Committee, Faculty of Medicine, Mashhad University of Medical Sciences, Mashhad, Iran; ^2^​ Antimicrobial Resistance Research Center, Mashhad University of Medical Sciences, Mashhad, Iran; ^3^​ Department of Microbiology and Virology, School of Medicine, Mashhad University of Medical Sciences, Mashhad, Iran; ^4^​ Laboratory of 22nd Bahman hospital, Khaf, Mashhad University of Medical Sciences, Mashhad, Iran; ^5^​ Department of Parasitology, 22nd Bahman hospital, Khaf, Mashhad University of Medical Sciences, Mashhad, Iran

**Keywords:** *Balantioides coli*, *Trichomonas vaginalis*, urinary balantiosis

## Abstract

**Background:**

Balantiosis, a rare zoonotic infection resulting in dysentery, is caused by the large ciliated protozoan parasite *Balantioides coli*. Humans acquire this organism via the faecal–oral route. Very rarely, extraintestinal infections can occur in the urinary tract system. There are very few case reports of urinary balantiosis in humans.

**Case presentation:**

Here, an interesting case of urinary balantiosis in a 35-year-old addicted woman with multiple health problems including spontaneous abortion and diabetes is reported. Her midstream urine sample, collected while all sterile precautions were being taken, demonstrated *B*. *coli* along with *Trichomonas vaginalis* and bacteria. *B. coli* was identified by its characteristic morphology and rapid rotary motility in the urinary tract, which is an abnormal site for invasion by this parasite.

**Conclusion:**

To the best of our knowledge, this is only the eighth case described in literature in which *B. coli* was detected in urine sediment; it is reported for its rarity and for future reference.

## Introduction

*Balantioides coli* (formerly *Balantidium coli*, synonym *Neobalantidium coli*) is a large ciliated protozoan parasite of low virulence, which causes balantidial dysentery in humans. It has a worldwide distribution, but the prevalence of its human infection is very low (Schuster & Ramirez-Avila, 2008). Balantiosis is a zoonotic disease acquired by humans via the faecal–oral route from its normal hosts, although it is an uncommon infection. Infection in humans is found mostly in tropical and subtropical regions and developing countries, especially in settings where water sources may be contaminated with porcine or human faeces and where hygiene is poor (Schuster & Ramirez-Avila, 2008). Human-to-human transmission may also occur, albeit rarely. Although *B. coli* infects a wide range of mammals, domestic pigs and wild boars are generally considered to be the most important natural reservoir for human balantiosis (Nakauchi, 1999). Thus, this infection has an increased prevalence in countries and communities that live in close contact with pigs. Because of the Islamic prohibition on eating pork, piggeries in Iran, a Muslim country, were closed after the 1979 Islamic revolution, and pig breeding and the consumption of pork was prohibited. Therefore, the pig can be excluded as a source of infection in Iran. However, there are still reports of this infection, which suggests that a different animal may be a reservoir host. The wild boar is found in rural areas of different parts of Iran and has close contact with farmlands and natural water sources. Additionally, some reports have shown that *B. coli* is found most frequently in Iranian wild boars. Therefore, they are probably the source of human infection in Iran, especially in rural areas where they roam and their faeces could contaminate food and water (Solaymani-Mohammadi *et al.*, 2005).

In humans, the caecum and colon are the natural habitats of this organism. After ingestion, *B. coli* can penetrate the mucosal layer of the large intestine and cause ulcers and balantidial dysentery in humans and other vertebrates (Schuster & Ramirez-Avila, 2008). In rare cases, especially in immunocompromised subjects, extraintestinal spread to the appendix (Dodd, 1991), liver (Kapur *et al.*, 2014), peritoneum (Ferry *et al.*, 2004), lung (Sharma and Harding, 2003) and genitourinary tract has also been reported (Rivasi and Giannotti, 1983; Koopowitz *et al.*, 2010). Genitourinary infections, including uterine infection, vaginitis and cystitis, are thought to occur via direct spread from the anal area (usually in females) or as secondary to a rectovaginal fistula created from infection with this organism (Umesh, 2007).

Clinical manifestation of balantiosis can take one of three forms: (i) asymptomatic or mild infection that presents with intermittent diarrhoea alternating with constipation; (ii) acute or fulminating forms presenting with stools containing mucus and blood, resembling *Entamoeba histolytica* dysentery, with severe dehydration and weight loss; in rare cases, fulminant ulceration with perforation of the colon may cause haemorrhage and even death; and (iii) chronic symptomatic infection, which is characterized by non-bloody diarrhoea alternating with constipation and non-specific abdominal pains (Bellanger *et al.*, 2013). Extraintestinal spread to the genitourinary tract has rarely been reported (Koopowitz *et al.*, 2010). Urinary balantiosis is thought to occur via direct spread from the anal area or a rectovaginal fistula created from infection with *B. coli* (Umesh, 2007). This paper reports a rare and interesting case of urinary balantiosis in an addicted, diabetic woman showing ciliated parasites along with *Trichomonas vaginalis* and bacteria in a midstream urine sample collected while all sterile precautions were being taken.

## Case report

A 35-year-old addicted woman suffering from diabetes and hypothyroidism was referred to the hospital emergency department with the chief complaint of spontaneous abortion and a temporary loss of consciousness. Abdominal examination of this patient revealed generalized tenderness. A complete haemogram and other laboratory tests (Table 1) revealed severe anaemia, biological inflammatory syndrome (C-reactive protein positive), hypernatremia, hypokalaemia, diabetes and an initial functional renal deficiency. Examination of the arterial blood gas revealed metabolic acidosis (Table 1).

**Table 1.** Laboratory test results of the patient

Laboratory test	Results	Clinical implications
**Haemogram tests**		
Total leukocyte count	17.5 × 10^3^ μl^− 1^	Leukocytos
Total platelet count	374 × 10^3^ μl^− 1^	
Haemoglobin level	8.9 g dl^− 1^	Anaemia
**Biochemistry tests**		
Serum Na^+^	147 mmol l^− 1^	Hypernatremia
Serum K^+^	2.9 mmol l^− 1^	Hypokalaemia
Blood sugar	520 mg dl^− 1^	Diabetes
Fasting blood sugar	210 mg dl^− 1^	
**Kidney function tests**		
Serum urea	17 mg dl^− 1^	Initial functional renal deficiency
Serum creatinine	1.8 mg dl^− 1^	
**Examination of arterial blood gas**		
pH	6.985	Metabolic acidosis
PaCO_2_	11.4 mmHg	
PaO_2_	78.9 mmHg	
Bicarbonate	2.7 mmol l^− 1^	

**Fig. 1.**
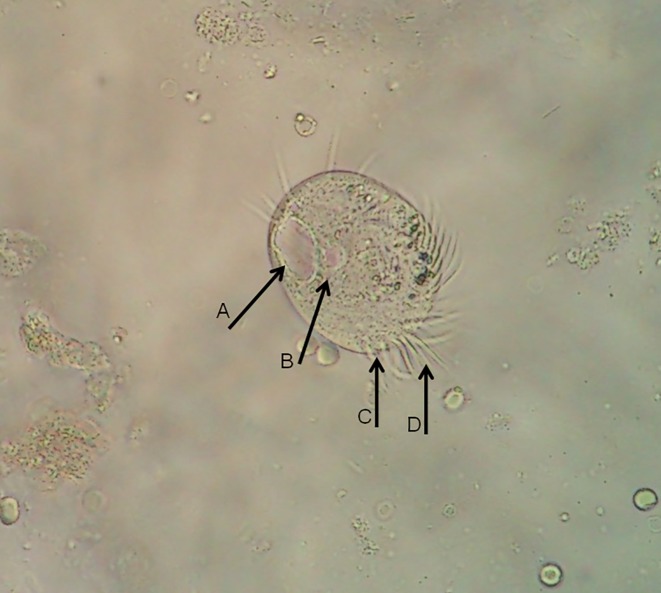
Trophozoite of *B. coli* with micronucleus, macronucleus and food vacuoles in urine sample. A, macronucleus; B, micronucleus; C, cytostome; D, cilia lining the mouth part, which appeared to be longer than the others (adoral cilia). Magnification, × 1000.

**Fig. 2.**
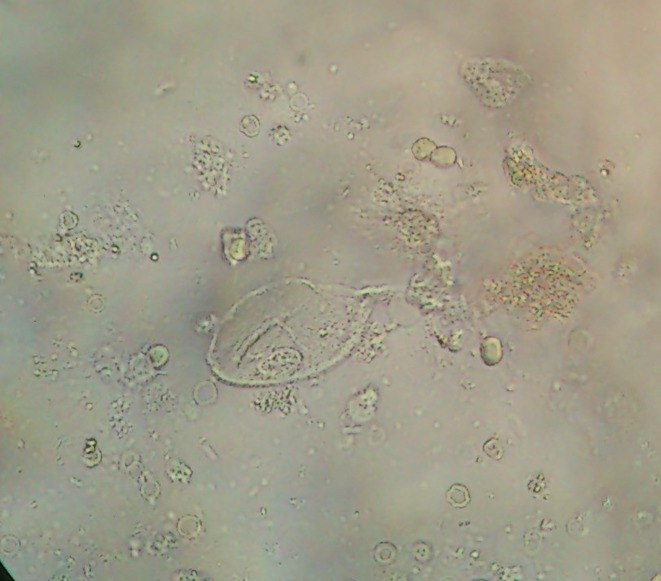
Trophozoites of *T. vaginalis* in the urine sediment. Magnification, × 1000.

The patient's fresh midstream urine sample was sent for macroscopic and microscopic examination. Its physical appearance was smoky and turbid, and a urine test strip revealed sugar and proteinuria. Samples were examined using the merthiolate–iodine–formaldehyde concentration method (Pomajbíková *et al.*, 2010). Microscopic examination of the sediment revealed a tubular cast, a few epithelial cells, amorphous urates, bacteria, haematuria with 50–60 red blood cells per high-power field and 20–25 pus cells per high-power field. The urine sample showed a few large ovoid-shaped ciliated parasites measuring approximately 61 × 51 μm. They had a rotary, boring motion and were moving very rapidly across the slide (Video S1, available in the online Supplementary Material), suggesting trophozoites of *B. coli* (Fig. 1). The trophozoite bodies were completely covered with short delicate cilia, all of uniform length, which maintained a constant synchronized motion. The cilia that lined the mouth part appeared to be longer than the others (adoral cilia). The organism had a mouth that was located at the pointed anterior end (cytostome) and a rounded posterior end (cytopyge). Several food vacuoles, a macronucleus, a micronucleus (next to the macronucleus) and a few ingested red blood cells were present within the cytoplasm. This morphology, the ciliary covering and its rotary motion were typical of *B. coli*. In the urine sediment, a few motile trophozoites of *T. vaginalis* were also seen (Fig. 2). Repeated midstream urine samples from the patient were also positive for similar organisms. The stool samples collected were negative for *B. coli* trophozoites or cysts, but mucosal clamps with many pus cells were seen. The patient was given tetracycline (500 mg four times a day) and metronidazole (750 mg three times a day) for a total of 7 days, and she gradually improved.

## Discussion

Human urinary balantiosis is a rare disease. Before 2015, there were only seven cases reported in literature: one from Iran (Maleky, 1998), one from Italy (Maino *et al.*, 2010) and five cases in India (Umesh, 2007; Basavraj *et al.*, 2012; Bandyopadhyay *et al.*, 2013; Khanduri *et al.*, 2014; Karuna & Khadanga, 2014).

In the case presented here, a 35-year-old female patient presented with diabetes and addiction, both of which can make a patient vulnerable to invasion by opportunistic microbes at any site. She denied direct contact with swine or wild boar, but had a history of eating the meat of hunted rabbit; thus, she might have been infected from contaminated rabbit meat, although there has so far been no report of human infection via consumption of rabbit meat in the world. It seems that more studies molecular and phylogenetic methods are required to determine the existence of *Balantioides*-like ciliates in rabbits (Pomajbíková *et al.*, 2013). On the other hand, the patient lives in a village and is in contact with wild animals, so it is not unreasonable to surmise that she may have consumed water or food contaminated with wild boar faeces containing *B. coli* cysts. After ingestion and excystation in the large intestine, the trophozoites may have invaded the colonic mucosa to enter the bloodstream and finally metastasized to the bladder or spread directly through the anal area. The patient also mentioned that she had had diarrhoea previously, but at the time of admission she had severe constipation, which implied the common phase of a parasite.

In conclusion, patients with uncontrolled diabetes are considered to be immunosuppressed due to the negative effect of elevated blood sugars on the immune system. Hyperglycaemia destroys overall immunity by various mechanisms and can lead to acidosis, which limits the activity of the immune system (Casqueiro *et al.*, 2012). Moreover, addictive drugs and opioids suppress the immune system, the body's innate defence against infections (Friedman *et al.*, 2003). Immunosuppressed patients appear to be less resistant to balantiosis. Thus, *B. coli* can become an opportunistic parasite in immunosuppressed, diabetic and addicted patients such as the present case, living in rural environments where wild boars roam and their faeces could contaminate food and water. There have been no more studies, however, to determine the prevalence of balantidia in immunocompromised individuals. Nevertheless, some extraintestinal infections have been reported previously in association with spread of *B. coli* to the lung (Anargyrou *et al.*, 2003), peritoneum (Ferry *et al.*, 2004) and genitourinary tract (Rivasi and Giannotti, 1983), especially in immunocompromised subjects. Thus, this case emphasizes the fact that *B. coli* should also be considered as a possible pathogen in immunocompromised patients with or without diarrhoea, even if they have no contact with pigs (Anargyrou *et al.*, 2003; Cermeño *et al.*, 2003; Yazar *et al.*, 2004). It should come in the differential diagnosis of these patients presenting with dysuria and haematuria. Using microscopic examination of fresh urine sediment, a microbiologist can easily diagnose this large parasite by its characteristic morphology and rapid rotary motility.

## Supplementary Data

14088 Supplementary Data
